# MTHFR, XRCC1 and OGG1 genetic polymorphisms in breast cancer: a case-control study in a population from North Sardinia

**DOI:** 10.1186/s12885-020-06749-w

**Published:** 2020-03-19

**Authors:** Matteo Floris, Daria Sanna, Paolo Castiglia, Carlo Putzu, Valeria Sanna, Antonio Pazzola, Maria Rosaria De Miglio, Francesca Sanges, Giovanna Pira, Antonio Azara, Emanuele Lampis, Antonello Serra, Ciriaco Carru, Maristella Steri, Flavia Costanza, Marco Bisail, Maria Rosaria Muroni

**Affiliations:** 1 Department of Biomedical Sciences, University of Sassari, Sassari, Italy; 2 Department of Medical, Surgical and Experimental Sciences, University of Sassari, Sassari, Italy; 3 Division of Medical Oncology, AOU Sassari, Sassari, Italy; 4 Unit of Occupational Medicine, AOU Sassari, Sassari, Italy; 5 Institute for Genetic and Biomedical Research, National Research Council (CNR), Monserrato, Cagliari, Italy; 6 LILT, Sassari, Italy

**Keywords:** Mediterranean population, MTHFR SNPs, XRCC1 SNPs, OGG1 SNPs

## Abstract

**Background:**

Despite conflicting results, considerable evidence suggests the association between single nucleotide polymorphisms in *MTHFR*, *XRCC1* and *OGG1* genes and, risk of developing breast cancer. Here a case-control study is reported, including 135 breat cancer patients and 112 healthy women, all representative of Northern Sardinian population.

**Methods:**

Polymerase chain reaction/restriction fragment length polymorphism method was used to determine the genotypes of five polymorphisms: *MTHFR* C677T (rs1801133) and A1298C (rs1801131), *XRCC1* Arg194Trp (rs1799782) and Arg399Gln (rs25487) and *OGG1* Ser326Cys (rs1052133). Allelic, genotypic and haplotype association analyses with disease risk and clinicopathological parameters were performed.

**Results:**

A nominally significant association with breast cancer risk was observed for *MTHFR* C677T polymorphism heterozygous genotype in the codominant model (OR: 0.57, 95% CI: 0.32–1.00, *p* = 0.049) and for Cys/Cys genotype of the *OGG1* Ser326Cys polymorphism in the recessive model (OR: 0.23, 95% CI: 0.05–1.11, *p* = 0.0465). No significant differences were found at genotype-level for A1298C polymorphism of the *MTHFR* gene and Arg194Trp and Arg399Gln of the *XRCC1* gene. Furthermore, the *OGG1* and *XRCC1* rs25487 polymorphisms were nominally associated with PgR, Her2 status and with sporadic breast cancer, respectively.

**Conclusions:**

Based on genetic characteristics of individuals included in this study, results suggest that *MTHFR* CT and *OGG1* Cys*/*Cys genotypes have a protective effect that may have an influence on breast cancer risk in a representative Northern Sardinian population.

## Background

Breast cancer (BC) is the most common malignancy and remains the main cause of cancer-related deaths in women worldwide [1]. Although mechanisms of breast carcinogenesis have not been completely elucidated, it is known that genetic factors can interact with numerous environmental factors thus determining an overall individual susceptibility. The study of BC susceptibility in isolated populations - where evolutionary forces and genetic features might have amplified the effect of specific risk alleles - offers the opportunity to shed further light on the pivotal role of genetic alterations in the occurrence of cancer. In such a context, the Mediterranean island of Sardinia shows a unique high incidence of several diseases with monogenic and multifactorial inheritance [2–4] and represents an important case-study population for human geneticists interested in inferring the complex mapping of traits and diseases. The relatively homogeneous genetic composition reported for Sardinian population has shown to be useful in depicting genetic factors of predisposition to BC [5–7]. A gap in BC incidence is observed in Italy between areas in northern Italy compared to central and southern areas; Sardinia shows the highest incidence rate (134,4 new cases/100.000 inhabitants) among southern regions, and the lowest among areas in northern Italy [8]. Here we reported a pilot case-control study carried out on a cohort of 135 northern Sardinian BC patients and 112 healthy controls, who were genotyped for polymorphisms in three restriction length molecular markers with frequency in Sardinian population > 5%. Specifically, the present study aimed to evaluate the role as risk factors of five genetic variants in three genes involved in methylation and DNA synthesis and in DNA repair mechanisms: the Methylenetetrahydrofolate reductase gene (*MTHFR*), the x-ray repair cross-complementing group 1 gene (*XRCC1*) and the 8-Oxoguanine glycosylase gene (*OGG1*). We also proposed to evaluate the possible association between these Single nucleotides polymorphisms (SNPs) of these genes and the main clinicopathological features.

The MTHFR is a key enzyme in the folate metabolic pathway and catalyses the conversion of 5,10-methylenetetrahydrofolate to 5-methyltetrahydrofolate, which is the circulating form of folate and the methyl donor in biological processes including methylation of proteins and nucleic acids. Furthermore, MTHFR plays an essential role in de novo synthesis of purines and pyrimidine nucleoside, but also in DNA biosynthesis, repair and maintenance of DNA stability. Two common polymorphisms in the *MTHFR* gene, C677T-rs1801133 and A1298C-rs1801131, have been associated with decreased enzyme activity and increased levels of plasma homocysteine [9–11]. The C677T allelic variant involves a cytosine to thymine substitution at position 677, a consequence of transformation from an alanine to a valine at codon 222 in the N-terminal catalytic domain, and it is correlated with increased thermolability and reduced enzyme activity. The A1298C allelic variant results into the change from a glutamic acid to alanine residue at codon 429 in the C-terminal regulatory domain of the protein but the reduction in enzymatic activity remains still controversial. Public eQTL data (GTEX catalog, release V8, accessed 26 Nov 2019), show that the rs1801133 minor allele causes a decreased expression of *MTHFR* gene in skin cells and cultured fibroblasts, while the minor allele of rs1801131 is associated with higher expression of the gene in many different tissues. However, an evident linkage between the polymorphic variants of the *MTHFR* gene and the increased risk of BC has not been clearly established. Functional relevance of rs1801133 has been confirmed by GWAS data about regulation of serum folate levels [https://www.ebi.ac.uk/gwas/variants/rs1801133] and other haematological traits (https://genetics.opentargets.org/variant/1_11796321_G_A), as supported by studies about high-altitude adaptation in Tibetans [www.ncbi.nlm.nih.gov/pubmed/28373541]; rs1801131 is associated with pressure related traits and hypertension (https://genetics.opentargets.org/variant/1_11794419_T_G).

The *XRCC1* gene is located on chromosome 19q13.2 and contains 17 exons that encodes a 70 kDa protein consisting of 633 amino acids. Shen et al. [12] have identified two polymorphisms resulting in nonconservative amino acid substitution variants in the *XRCC1* gene. In this study C → T substitution at codon 194 in exon 6 (Arg to Trp, rs1799782) and G → A substitution at codon 399 in exon 10 (Arg to Gln, rs25487) were analysed. The Arg194Trp polymorphism is close to a modified residue (phosphothreonine in position 198), and it is located in the linker region between the NH2-terminal domain and the BRCT1 (BRCA1 C-terminus) domain (positions 315–403); the Arg399Gln polymorphism occurs in important biologically interaction domains with PARP (poly ADP-ribose polymerase). The Trp derived allele has been associated with an increased Base Excision Repair (BER) activity, while the 399Gln allele with its reduction [13]. Public eQTL data show that the effect of rs25487 on the expression of *XRCC1* is strictly tissue dependent, showing instead for example opposite effects of the minor allele in nerve tibial cells and testis derived cells; the rs1799782 minor allele causes a minor gene expression in thyroid cells.

*OGG1* gene, which is located on chromosome 3p26.2 and also involved in BER, encodes a DNA glycosylase and apurinic/apyrimidinic lyase activities to excise 8-oxoguanine (8-OH-G), a mutagenic base which occurs as a result of Reactive oxigen species (ROS) exposure. Ser326Cys in exon 7 (rs1052133) is the most frequent and most studied polymorphism in the *OGG1* gene. Amino acid replacement of serine with cysteine causes a reduced OGG1 DNA repair activity [14]. The effect on gene activity is confirmed by eQTL data, with the minor allele being associated with lower expression of *OGG1*.

These polymorphisms have been previously correlated with various cancer types [15–20] and differences in their allelic variant effects have been reported for different ethnic groups [21]. However, they have never been investigated so far within the Sardinian population with the scope to find, if any, a possible evident correlation between polymorphisms occurrence and BC risk, clinicopathological traits and lifestyle exposures.

## Methods

### Sampling plan

In this case-control study, conducted between 2017 and 2019, a total of 247 unrelated women were included: 135 BC patients enrolled at the Medical Oncology Unit in Sassari University Hospital and a control group consisting of 112 healthy women, who had never previously been affected by any tumour, enrolled at the Unit of Occupational Medicine of the University of Sassari.

A further control group, formed by 109 women who had voluntarily accessed to Lega Italiana Lotta contro i Tumori (LILT, Sassari) prevention facilities for investigations, was also analyzed. However, LILT volunteers’ sample was then excluded from the study as a self-selection bias was highlighted (data not shown), being such a group of individuals exposed to a higher level of familiarity compared to the other healthy controls.

In order to recruit only individuals representative of Sardinian population, the participants were selected on the basis of their surname (which had to be typically Sardinian) and place of birth of their grandparents (which had to be located in Northern Sardinia). All women taking part to the study were asked to fill in a questionnaire regarding a wide range of all known risk factors for BC development: i.e. demographic characteristics, body weight and Body mass index (BMI), reproductive-hormone profile, family history of cancer, passive and active cigarette smoking (pack-years were calculated as years smoked multiplied by the current number of cigarettes smoked per day divided by 20) [17], lifetime alcohol use (only the frequency was evaluated and not the quantity introduced), physical activity (at least two to three times a week), fruit and vegetable intake (classified as high at least two portions per day), diet composition and more. In completing the questionnaire regarding lifestyle, the participants were invited to consider the widest possible time span and not only the period close to the enrolment.

The questionnaire collected all information needed to identify the familial forms of BC compared to sporadic ones. In summary, the inclusion criteria adopted for identified family forms were: one case with BC diagnosis before 39 years of age and/or bilateral diagnosis of BC before the age of 40 in two first or second degree relatives; presence of 3 or more cases of BC in first or second degree relatives in the same side of the family tree; cases of ovarian cancer; male BC cases; presence of multiple primary cancers in any organ in the same individual or family [22].

### Polymorphisms genotyping

EDTA blood samples were obtained from all study participants at the moment of enrolment and were stored at − 20 °C until further use.

Genomic DNA was extracted from 200 μl of peripheral blood by QIAmp DNA Blood Mini Kit (Qiagen, Germany) following manufacturer’s instructions.

The following reported allelic variants for three genes were investigated by Polymerase chain reaction/restriction fragment length polymorphism (PCR-RFLP) assay: *MTHFR* C677T (rs1801133) and A1298C (rs1801131), *XRCC1* Arg194Trp (rs1799782) and Arg399Gln (rs25487) and *OGG1* Ser326Cys (rs1052133).

**Fig. 1:**
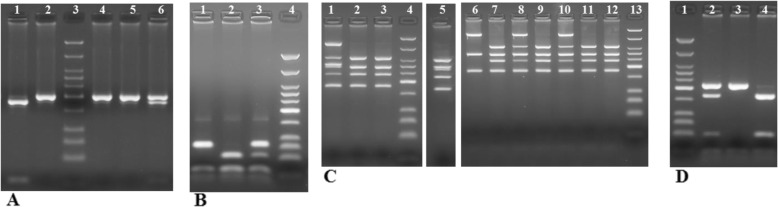
Electrophoretic separation of *MTHFR*, *XRCC1* and *OGG1* digestion in 3% Metaphor gel. Marker of molecular weight: Low Molecular Weight DNA Ladder (NEB). **a***MTHFR* C677T: lane 1 TT (178, 23 bp), lanes 2, 4, 6 CC (201 bp), lane 6 CT (201, 178, 23 bp), lane 3 marker molecular weight; **b***MTHFR* A1298C: lane 1 CC (84, 31, 30, 28, 18 bp), lanes 2 AA (56, 31, 30, 28, 18 bp), lane 3 AC (84, 56, 31, 30, 28, 18 bp), lane 4 marker molecular weight; **c***XRCC1* Arg194Trp (**bold**) and Arg399Gln PCR-multiplex: lane 1 **arg/trp**-arg/gln (615, 374, **313**, **292**, 241, **174** bp), lanes 2, 3, 7, 9, 11, 12 **arg**/**arg**-arg/arg (374, **292**, 241, **174** bp), lane 5 **arg**/**trp**-arg/arg (374, **313**, **292**, 241, **174** bp), lane 6 **arg**/**arg**-gln/gln (615, **292**, **174** bp), lanes 8,10 **arg**/**arg**-arg/gln (615, 374, **292**, 241, **174** bp), lanes 4, 13 marker molecular weight. **d**. *OGG1* Ser326Cys: lane 1 marker molecular weight, lane 2 ser/cys (213, 164, 49, 21 bp), lane 3 ser/ser (213, 21 bp), lane 4 cys/cys (164, 49, 21 bp)

*MTHFR* C677T and A1298C: genotyping was performed as previously reported [23] (Fig. 1a-b).

*XRCC1* Arg194Trp and Arg399Gln: a multiplex PCR was used to amplify 491 and 615 bp fragments containing the 194 and 399 polymorphisms respectively [24]. PCR was performed in a total reaction volume of 25 μl containing 100 ng DNA, 1x PCR buffer, 2 mM of MgCl_2_, 0.2 mM of each dNTP, 1.5 units of Taq Gold DNA polymerase (Applied Biosystems, USA), 0.6 μM for codon 194 of each primer (5′ *GCC CCG TCC CAG GTA* 3′ and 3′ *AGC CCC AAG ACC CTT TCA* CT 5′), and 0.8 μM for codon 399 (5′ *TTG TGC TTT CTC TGT GTC CA* 3′ and 3′ *TCC TCC AGC CTT TTC TGA TA* 5′). Thermal cycling conditions consisted of an initial denaturation at 95 °C for 5 min, followed by 35 cycles of 95 °C for 45 s, 56.4 °C for 45 s, 72 °C for 60 s with a final extension at 72 °C for 5 min. The Arg alleles at codons 194 and 399 create *Msp*I restriction sites. The multiplex PCR products were digested with *Msp*I (New England Biolabs, USA) in a volume of 25 μl at 37 °C for 3 h. Among the genotypes for codon 194, the Arg/Arg resulted in 292, 174 and 21 bp, Arg/Trp in 313, 292, 174 and 21 bp, Trp/Trp in 313 and 174 bp digestion products. A 174 bp fragment was present in all the samples because of an invariant *Msp*I site that was used as an internal control for complete digestion. Among the genotypes for codon 399, the Arg/Arg resulted in 374 and 241 bp, Arg/Gln in 615, 374 and 241 bp, Gln/Gln in 615 bp digestion products. (Fig. 1c).

*OGG1* Ser326Cys: PCR was performed with the final volume of 20 μl of reaction mixture containing 100 ng DNA, 5x PCR buffer, 0.2 mM of each dNTP, 2.5 units of Q5 Hot Start High-Fidelity DNA Polymerase (New England Biolabs, USA) and 0.6 μM of each primer (5′ *CCC AAC CCC AGT GGA TTC TCA TTG* C 3′ and 5′ *GGT GCC CCA TCT AGC CTTGCG GCC CTT* 5′)*.* Thermal cycling conditions consisted of an initial denaturation at 98 °C for 30 s, followed by 35 cycles of 98 °C for 30 s, 67 °C for 30 s, 72 °C for 30s with a final extension at 72 °C for 5 min. The 234 bp PCR product was digested using *Fnu4HI* (New England Biolabs, USA) in a volume of 25 μl for 3 h at 37 °C. A mismatch was incorporated in the reverse primer to create an invariant restriction site of 21 bp which produced a 21 bp fragment in all the samples [25]. The C → G cysteine variant creates a *Fnu4HI* restriction enzyme site and produces fragments of 213, 164, 49 and 21 bp (Fig. 1d). All the PCR products were electrophoresed in a 3% Metaphor agarose gel stained with ethidium bromide and visualized by UV transillumination.

### Statistical analysis

Minor allele frequency of the five polymorphisms here considered has been verified in the public resource SardiNIA Pheweb (https://pheweb.irgb.cnr.it/).

The allelic status of each individual was inferred based on the SNPs analysis performed by PCR-RFLP.

Agreement on genotype frequencies to Hardy-Weinberg equilibrium (HWE) expectation was assessed independently among controls for each SNP, using a 1-degree-of-freedom chi-square test.

Student’s t-test and Fisher’s exact test were used to evaluate the differences in descriptive variables at diagnosis between the cases and controls, such as age or BMI.

For each SNP, the genotypic and allelic association were tested considering multiple inheritance models (additive, dominant, codominant and over-dominant, and recessive), using a custom R script, to compare BC patients with the group of healthy controls enrolled in the present study. The R package *fsmb* was used to calculate odds ratios (OR) and 95% confidence intervals (95% CI) according to Woolf method. When a 0 count was observed a Gart-adjusted logit interval was calculated.

Before association, potential confounders (as age and BMI) were tested by including them as covariates in the association models; none of them showed a significant impact, so no covariate was added in the analyses.

Haplotype analysis was restricted to polymorphisms located on the same chromosome: i) the haplotype rs1801133/rs1801131 (*MTHFR*) and ii) the haplotype rs1799782/rs25487 (*XRCC*1). Measures of Linkage disequilibrium (LD) between each pair of SNPs (D’ and r^2^ statistics) and haplotype reconstruction were obtained in 1000 genomes European population with the LDpair web tool (https://ldlink.nci.nih.gov/?tab=ldpair). The most common haplotype was selected as the reference. OR and 95% CI were calculated to estimate the degree of the association between haplotypes and the risk of breast cancer.

A threshold of *p* ≤ 0.05 was used to assess statistical significance for each genetic association. A Bonferroni correction was also applied to adjust for multiple testing, obtaining a significant threshold of *p* < 0.0027 for differences in descriptive variables at diagnosis between the cases and controls (Table 1, numb. of tests = 18) and *p* < 0.01 for association analyses (Suppl. Table 1A, numb. tests = 5).

**Table 1:** Descriptive questionnaire data of cases and controls

Variables	Cases X	Cases n.	Cases %	Contr. X	Contr. n.	Contr. %	***P*** value
**Age**	57.00			50.03			**9.0E-07**^a^
**Height**	159.24			160.50			0.1
**Weight**	64.50			60.20			**0.002**^a^
**BMI**	25.40			23.40			
< 25		72	53.33		80	71.43	**0.004**
25–30		38	28.15		25	22.32	
≥ 30		25	18.52		7	6.25	
**Age at menarche**	12.44			12.72			0.1
7–10		12	8.89		8	7.14	0.7
11–13		90	66.67		72	64.28	
14–17		33	24.44		32	28.58	
**Menopause**							
natural menopause		62	45.93		55	49.11	0.6
age	49.90			48.60			0.1
HRT regimen		11	17.74		8	14.54	0.6
**Parity**		97	71.85		71	63.39	0.2
first full-term pregnancy	27.59			29.56			0.1
number of live birth	2.05			1.82			0.1
**Breastfed**		78	80.41		63	88.73	0.2
months of breastfeeding	11.83			12.72			0.9
**Oral contraceptive use**		78	57.77		87	77.67	**0.001**^a^
duration of employment:							
≤ 5 y		20	25.64		33	37.93	0.2
6–14 y		35	44.87		33	37.93	
≥ 15 y		23	29.49		21	24.14	
**Benign breast lesions**		29	21.48		12	10.71	**0.03**
**BC family**							
in first degree relatives	1.34			0.74			**8.8E-05**^a^
0 cases		47	34.81		62	55.36	**4.0E-04**^a^
1–2 cases		58	42.96		42	37.50	
≥ 3		30	22.22		8	7.14	
**Tumor family**							
in first degree relatives:	3.42			2.81			**0.03**
0–2 cases		51	37.78		57	50.90	0.05
3–5		57	42.22		43	38.39	
≥ 6		27	20.00		12	10.71	
**Alcohol intake**							
daily-often		31	22.96		13	11.61	**0.03**
occasionally- nondrinkers		104	77.04		99	88.39	
**Smoking status** (smokers at recruitment)		19	14.07		18	16.07	0.7
Smokers (at recruitment+ex smokers)		68	50.37		56	50.00	0.2
cigarette packs/year:							
< 10		30	44.12		25	44.64	0.3
10–19		16	23.53		19	33.93	
≥ 20		22	32.35		12	21.43	
passive smoke:		100	74.07		87	77.68	**5.0E-04**^a^
little		22	22.00		41	47.13	
moderately		46	46.00		27	31.03	
strong		32	32.00		19	21.84	
**Osteoporosis**		41	30.37		22	19.64	0.06
**Chemical exposure**		43	31.85		18	16.07	**0.04**
**Sport activity** (2–3 times a week)		45	33.33		43	38.39	0.4
**Adolescence sport activity**		36	26.66		56	50.00	**2.0E-04**^a^

^a^Significant after Bonferroni correction

To assess whether the cohort from Northern Sardinia enrolled in this study may be considered as representative of the whole Sardinian population and consequently to evaluate the reliability of our sample in being representative of whole Sardinian population genetic variation, we performed a 1-df chi square test to compare allelic frequencies between our groups and the whole Sardinia population.

The public eQTL data used for the analyses described in this manuscript were obtained from the GTEx Portal on 12/01/2019 (https://gtexportal.org/home/).

To evaluate the interaction between personal habits and SNPs with significant association with BC risk, a logistic regression model was performed where the multiplicative term of alcohol consumption, dietary folate intake and smoke and the SNPs were considered.

## Results

### Patient’s lifestyle and behaviour characteristics

Table 1 summarizes data emerged from the questionnaire analysis that were related to demographic, hormonal-reproductive and lifestyle characteristics of all participants in our case-control study. The mean age of cases and controls at the time of enrolment was 57.0 and 50.03, respectively (*p* = 9 × 10^− 7^). No significant differences emerged between BC patients and healthy women regarding menarche and menopausal age, parity and breastfeeding. There was an increase in BMI in patients (*p* = 0.004) compared to controls, and an increase in the use of oral contraceptives in the control group compared to cases (*p* = 0.001). The BC patients had benign lesions of the breast (*p* = 0.03) and a familiarity of BC (*p* = 8.8 × 10^–5)^ and tumours in general (p = 0.03) higher than controls. A greater number of affected women claimed to take alcohol daily/often (*p* = 0.03), to be subjected to passive smoking (*p* = 5 × 10^− 4^) and to be exposed to environmental toxic substances for work or other reasons (*p* = 0.04). Finally, a greater amount of practice in competitive physical activity during adolescence was significantly highlighted in the control group compared to the cases (*p* = 2 × 10^− 4^). There were no significant differences in the introduction of fruit and vegetable in the diet, as well as white meat, milk, eggs, fish, etc. While it emerged that in women affected by BC the following were introduced more frequently in their diet: red meat, cured meat, cheese, bread, pasta, potatoes, legumes, butter and lard and less frequently cereals (data not shown).

### Clinicopathological data related to BC patients

Data on clinicopathological characteristics of BC patients enrolled in this study are shown in Table 2. Most of the tumours were invasive ductal carcinoma (IDC) (85.18%) and early stage tumours (65.91%). According to the Nottingham prognostic index for BC combined to the histology grade, 12 patients were classified as G1 (8.89%), 79 as G2 (58.52%) and 43 as G3 (31.85%). Finally, molecular subtypes of the 135 BC were: 57 *Luminal A* (42.22%), 21 *Luminal B-like Her-2 neg* (15.55%), 21 *Luminal B-like Her-2 pos* (15.55%), 21 *Her-2 overexpressing* (15.55%) and 15 *Triple negative* (11.11%). The table also shows data concerning age at diagnosis and laterality (43.70 and 56.30% respectively).

**Table 2:** Clinicopathological features of BC patients

Variables of BC (n. = 135)		n.	(%)
**Age at diagnosis** (mean years)		52.15	
≤ 40		22	16.30
> 40		113	83.70
**Family BC**			
Sporadic		76	56.30
Familial		59	43.70
**Laterality**			
Bilateral		11	8.15
Unilateral		124	91.85
**Menopausal status** (at diagnosis)			
premenopausal		72	53.33
postmenopausal		63	46.66
**Histologic type**			
IDC		115	85.18
ILC		10	7.40
D or L in situ		7	5.18
Other		3	2.22
**Histologic grade**			
G1		12	8.89
G2		79	58.52
G3		43	31.85
missing		1	0.74
**Hormonal receptor**			
**ER**	pos	98	72.59
	neg	37	27.41
**PgR**	pos	91	67.40
	Neg	44	32.60
**Her-2**			
pos		42	31.11
neg		88	65.18
missing		5	3.70
**Ki67**			
≤ 30%		103	76.30
> 30%		31	22.96
missing		1	0.74
**Tumor size (T)**			
T0-Tis		7	5.18
T1-T2		96	71.11
T3-T4		32	23.70
**Lymph nodes grade (N)**			
N0		59	43.70
N1		40	29.62
N2		19	14.08
N3		7	5.18
Nx		10	7.41
**Distant Metastasis**			
M0		111	82.22
M1		22	16.30
missing		2	1.48
**Clinical stages**			
0		12	8.88
I		43	31.85
II		34	25.18
III		19	14.10
IV		21	15.55
missing		6	4.44
**Luminal A**		57	42.22
**Luminal B-like Her-2 neg**		21	15.55
**Luminal B-like Her-2 pos**		21	15.55
**Her-2 overexpressing**		21	15.55
**Triple negative**		15	11.11

As regards the 59 familial BC reported in Table 2, a total of eleven families (8.15%) were reported with cases of BC insurgents at a young age and concomitant presence of cases of ovarian cancer, two families (1.50%) with cases of BC in the male, 26 (19.25%) families with three cases of BC, twenty-six (19.25%) with four cases and nine (6.66%) with five or more cases. Finally, the highly familiar BC had an average of BC cases of 4.82 and tumours in general of 2.06 compared to 2.47 and 1.93 of sporadic BC, respectively (data not shown).

### Association with disease risk

The distribution of allelic frequencies in the whole Sardinian population [26–28] for the five SNPs here considered, is reported in Table 3.

**Table 3:** Genotype and allele distribution of *MTHFR*, *XRCC1* and *OGG1* polymorphisms

Gene (SNP)	Model	Genotype	Cases n.135 (n. %)		Controls n.112 (n. %)		OR (95% CI)	p
***MTHFR*(rs1801133)** **Ref. Allele = C** **Alt. Allele = T**	**Codominant**	C/C	53	39.25	31	27.68	1.00	
		C/T	60	44.44	62	55.36	0.57 (0.32–1)	***0.049***
		T/T	22	16.30	19	16.96	0.68 (0.32–1.44)	0.31
	**Dominant**	C/C	53	39.25	31	27.68	1.00	
		C/T-T/T	82	60.74	81	72.32	0.59 (0.35–1.02)	0.06
	**Recessive**	C/C-C/T	113	83.70	93	83.03	1.00	
		T/T	22	16.30	19	16.96	0.95 (0.49–1.87)	0.89
	**Overdominant**	C/C-T/T	75	55.60	50	44.64	1.00	
		C/T	60	44.44	62	55.36	0.65 (0.39–1.07)	0.09
	**Allele**	C	166	61.48	124	55.36	1.00	
		T	104	38.52	100	44.64	0.78 (0.54–1.11)	0.17
	**Hardy Weinberg eq.**		*p* = 0.47		*p* = 0.25			
***MTHFR*(rs1801131)** **Ref. Allele = A** **Alt. Allele = C**	**Codominant**	A/A	66	48.88	56	50.00	1.00	
		A/C	55	40.74	49	43.75	0.95 (0.56–1.61)	0.86
		C/C	14	10.37	7	6.25	1.7 (0.64–4.5)	0.28
	**Dominant**	A/A	66	48.88	56	50.00	1.00	
		A/C-C/C	69	51.11	56	50.00	1.05 (0.63–1.73)	0.86
	**Recessive**	A/A-A/C	121	89.63	105	93.75	1.00	
		C/C	14	10.37	7	6.25	1.74 (0.68–4.46)	0.25
	**Overdominant**	A/A-C/C	80	59.26	63	56.2	1.00	
		A/C	55	40.74	49	43.75	0.88 (0.53–1.47)	0.63
	**Allele**	A	187	69.26	168	75.00	1.00	
		C	83	30.74	63	28.12	1.18 (0.8–1.74)	0.39
	**Hardy Weinberg eq.**		*p* = 0.69		*p* = 0.49			
***XRCC1*(rs1799782)** **Ref. Allele = C** **Alt. Allele = T**	**Codominant**	C/C	128	94.81	102	91.07	1.00	
		C/T	7	5.18	10	8.93	0.56 (0.21–1.52)	0.25
	**Allele**	C	263	97.40	214	95.53	1.00	
		T	7	5.18	10	4.46	0.57 (0.21–1.52)	0.26
	**Hardy Weinberg eq.**		*p* = 1.00		p = 1.00			
***XRCC1*(rs25487)** **Ref. Allele = G** **Alt. Allele = A**	**Codominant**	G/G	64	47.41	53	47.32	1.00	
		G/A	55	40.74	43	38.39	1.06 (0.62–1.82)	0.83
		A/A	16	11.85	16	14.28	0.83 (0.38–1.81)	0.64
	**Dominant**	G/G	64	47.40	53	47.32	1.00	
		G/A-A/A	71	52.59	59	52.68	1 (0.6–1.65)	0.99
	**Recessive**	G/G-G/A	119	88.15	96	85.71	1.00	
		A/A	16	11.85	16	14.28	0.81 (0.38–1.7)	0.57
	**Overdominant**	G/G-A/A	80	59.26	69	61.61	1.00	
		G/A	55	40.74	43	38.39	1.1 (0.66–1.84)	0.71
	**Allele**	G	183	67.78	149	66.52	1.00	
		A	87	64.44	75	33.48	0.94 (0.65–1.38)	0.77
	**Hardy Weinberg eq.**		*p* = 0.56		*p* = 0.14			
***OGG1*(rs1052133)** **Ref. Allele = C** **Alt. Allele = G**	**Codominant**	C/C	87	64.44	70	62.50	1.00	
		C/G	46	34.07	35	31.25	1.06 (0.62–1.82)	0.84
		G/G	2	1.48	7	6.25	0.23 (0.05–1.14)	0.05
	**Dominant**	C/C	87	64.44	70	62.50	1.00	
		C/G-G/G	48	35.55	42	37.50	0.92 (0.55–1.55)	0.75
	**Recessive**	C/C-C/G	133	98.52	105	93.75	1.00	
		G/G	2	1.48	7	6.25	0.23 (0.05–1.11)	***0.0465***
	**Overdominant**	C/C-G/G	89	65.92	77	68.75	1.00	
		C/G	46	34.07	35	31.25	1.14 (0.67–1.94)	0.64
	**Allele**	C	220	81.48	175	78.12	1.00	
		G	50	18.52	49	21.87	0.81 (0.52–1.26)	0.35
	**Hardy Weinberg eq.**		*p* = 0.24		p = 0.4			

None of the 1-df chi square tests used to compare allelic frequencies between our groups and the whole Sardinian population showed statistical significance. All SNPs were successfully genotyped in all participants to the present study and were in accordance with HWE in healthy controls (*p* > 0.05).

Using the chi-square test, the association between the SNPs tested and the BC risk under five gene models (codominant, dominant, recessive, overdominant and additive) was analysed. Minor allele frequencies in cases and controls were showed.

Genotype analysis of *MTHFR* polymorphisms showed that CT genotype frequency of rs1801133 polymorphism revealed a nominal association with lower risk of BC in the codominant model (CT vs. CC, OR: 0.57, 95% CI: 0.32–1.00, *p* = 0.049). This trend was confirmed by dominant and overdominant models (*p* = 0.055 and 0.088 respectively).

Furthermore, the analysis of combined effect of C677T and A1298C polymorphisms showed that no subject presented with homozygous mutant allele in both polymorphic sites.

Likewise, no significant effect of BC susceptibility was highlighted in this study for SNPs studied in *XRCC1*. No homozygous case was found neither in Arg194Trp nor in concurrent heterozygous allele Trp with Arg399Gln homozygous mutant.

Furthermore, the GG genotype frequency of *OGG1*-rs1052133 polymorphism revealed a nominal association with lower risk of breast cancer in the recessive model (GG vs. CC/CG, OR: 0.23, 95% CI: 0.05–1.11, *p* = 0.0465, with the trend confirmed in the codominant model for GG genotype (*p* = 0.05).

Association analysis between the risk of BC and haplotypes of *MTHFR* gene (rs1801133 - rs1801131) and *XRCC1* gene (rs1799782 - rs25487) were also performed. Haplotypes were reconstructed from the 1000G data and results of their distribution among BC cases and controls were summarized in Table 4. The pairwise LD is given for each pair of SNPs, and r^2^ measurements indicate that LD is low. Among the *MTHFR* haplotypes, rs1801133-T / rs1801131-A is the most frequent in both cases and controls, while rs1801133-T / rs1801131-C is absent (Table 4). Among the *XRCC-1* haplotypes, rs1799782 -C / rs25487 -G is the most frequent, while rs1801133-T / rs1801131-A is absent. Overall, none of the considered haplotypes is significantly associated with BC risk.

**Table 4:** Aplotype frequencies of *MTHFR* and *XRCC1* in BC patiens and controls D’ and r2 statistics calculated in 1000 genomes European population

Gene	Haplotype		Cases	Controls	OR (95% CI)	p
***MTHFR*** **D’ = 0.9999** **r2 = 0.262**	**rs1801133**	**rs1801131**				
	T	A	95 (38.52%)	110 (44.64%)	1.00	
	C	C	77 (31.11%)	70 (28.12%)	1.27 (0.83–1.95)	0.27
	C	A	75 (30.37%)	67 (27.23%)	1.30 (0.84–1.99)	0.24
	T	C	0.00	0.00	–	–
***XRCC1*** **D’ = −0.9997** **r2 = 0.031**	**rs1799782**	**rs25487**				
	C	G	160 (64.81%)	153 (62.05%)	1.00	
	C	A	81 (32.59%)	83 (33.48%)	0.92 (0.63–1.34)	0.67
	T	G	6 (2.59%)	11 (4.46%)	0.52 (0.19–1.45)	0.21
	T	A	0.00	0.00		

### Association between genotypes, clinical features and lifestyles

In this study the possible relationship between some clinicopathological parameters such as estrogen receptor (ER) and progesterone receptor (PgR) status, Her-2 and Ki67 expression and lymph node involvement (Suppl. Table 1A) and other parameters such as age at diagnosis, BMI, pre/post menopause diagnosis and family history of BC cases and the distribution of SNP genotypes was explored (Suppl. Table 1B).

Regarding the clinical features, only the *OGG1*-rs1052133 polymorphism is associated at the nominal level with PgR status: G allele is significantly more frequent among PgR- cases (allelic model: OR: 0.09, 95% CI: 0.04–2.04, *p* = 0.0439). Furthermore, it is significantly associated with Her2 status, being more frequent among Her2+ cases (recessive model: OR: 10.92, 95% CI: 0.51–232.81, *p* = 0.0399). None of these associations is confirmed after Bonferroni correction (Table 5).

**Table 5:** Association of OGG1 polymorphisms and PgR, Her-2 status in BC patients

Variable	Model	***OGG1*** rs1052133	OR (95% CI)	p
**PgR+/PgR-** **(n. 90/45)**	**Recessive**	C/C-C/G 90/43	1.00	–
		G/G 0/2	0.09 (0.04–2.04)^a^	***0.0439***
**Her2+/Her2 (n. 42/88)**	**Recessive**	C/C-C/G 40/88	1.00	–
		G/G 2/0	10.92 (0.51–232.81)^a^	***0.0399***

^a^Gart adjusted logit interval

Moreover, a nominal association of the *XRCC1*-rs25487 polymorphism was found with family history of BC, with G/A genotype associated in both the codominant and overdominant models (OR: 2.19; 95% CI: 1.04–4.62, *p* = 0.0393 and OR: 2.16; 95% CI: 1.06–4.41, *p* = 0.0337) respectively, no other significant association was found with the other clinicopathological features (Table 6).

**Table 6:** Association of *XRCC1* polymorphism rs25487 with BC family history status in BC patients

Variable	Model	***XRCC1*** (rs25487)	OR (95% CI)	p
**BC family history Spor/Famil** **(n.76/59)**	**Codominant**	G/G 31/33	1.00	–
		G/A 37/18	2.19 (1.04–4.62)	***0.0393***
		A/A 8/8	1.06 (0.36–3.18)	0.91
	**Overdominant**	G/G-A/A 39/41	1.00	–
		G/A 37/18	2.16 (1.06–4.41)	***0.0337***

We also explored the effect of polymorphisms in relation to smoking status (67 current smokers+ex-smokers versus 68 never smokers), alcohol intake (89 daily-often intake versus 46 non-drinkers) and fruit and vegetables intake (88 high versus 47 low intake, where intakes are classified as high if the individual consumed at least two portions of fruit and vegetables per day). It was not found any significant evidence of interaction between *MTHFR*-rs1801133 and *OGG1*-rs1052133 (SNPs with significant association with BC risk) polymorphisms and these lifestyles in cases and controls (data not shown).

## Discussion

BC is a complex and heterogeneous disease in which many genetic factors and lifestyle-related factors are combined, resulting into a multifactorial aetiology. Through the questionnaires we aimed to investigate the various risk factors and the effects of changes in lifestyle on mammary carcinogenesis.

In our study no significant differences between BC patients and controls were found in regards with hormonal-reproductive factors. However, the stratification in age groups of the menarche onset and the duration of oral contraceptive use shows a tendency towards an increased risk in the case of early onset of menarche and prolonged use of oral contraceptives, respectively. Similarly, a slight trend is observed for the average age of menopause and hormone replacement therapy regimen, while in our sample parity and breastfed are not aligned with most of the results in the literature [29–32]. Furthermore, our case-control study has confirmed that increase in BMI, the previous onset of benign lesions and genetic factors determining a high familiarity of BC and other tumors act as strong risk factors.

Several strong epidemiological and clinical studies support the correlation between mammary tumorigenesis, tumour progression and obesity. The main mechanisms underlying this link are capacity of conversion of androgenic precursors to estrogen through peripheral aromatization in adipose tissue, the alteration of physiological levels of insulin, insulin-like growth factor (IGF), adipokines, changes in the adipose tissue microenvironment and chronic low-grade inflammation [33–38]. Juarez-Cruz JC et al. [39] have recently shown that the leptin, a hormone secreted by adipocytes, through the FAK and Src kinases activation, contributes to BC progression. Physical activity is closely related to obesity. A meta-analysis study investigating the relationship with BC risk showed a 20% reduction if physical activity is performed in adolescence and within 24 years old [40], by delaying the onset of menarche and reducing the bioavailable hormones levels [41]. Our study has significantly confirmed these data in relation to obesity and physical activity.

In our case-control study we found a significant increase in the frequency of alcoholic drink intake in affected women compared to controls (daily-often 22.96% against 11.61 *p* = 0.03 respectively), even though quantity was not evaluated. Over 100 studies support a positive association between alcohol consumption and BC risk in all age groups. Alcohol increases the risk of BC in a dose-dependent manner, but unfortunately the awareness of alcohol as a risk factor is low as well as the ability of women to estimate the amount of alcohol contained in the drinks taken daily. It is clear that information campaigns are needed on this topic and other modifiable risk factors [42]. No difference was found between the two groups for what concerns fruit and vegetables intake.

Our findings revealed that affected women are strong smokers, in fact 32.35% of them have a packs/year value≥20 compared to 21.43% in controls. In addition, a strong impact of exposure to passive smoking emerged, in agreement with other studies that showed that exposure to passive smoking contributes to an increased risk in particular of Er+/PgR+ BC [43]. Finally, 31.85% BC women compared to 16.07% of controls, reported having come into contact throughout their lifetime with chemicals such as paints, solvents, pesticides, herbicides, environmental pollutants, textile industry products, etc.

Often the questionnaire procedure can produce selection bias that can, however, be minimized when the study is appropriately conducted. All women participating to our study completed the questionnaire after receiving the necessary information from the same operator. In completing the questionnaire regarding lifestyle, the participants were invited to consider the widest possible time span and not their conduct in the period close to enrolment. We noticed that many women tended to minimize, perhaps for cultural reasons, some factors such as alcohol consumption, the use of oral contraceptive and other avoidable factors, whose role in cancerogenesis is known, as the excessive introduction of foods such as cold cuts, red meat, dessert. Therefore, we believe that these data could be underestimated. On the contrary, the affected women showed excessive attention and evaluation towards possible contacts with chemicals.

In addition to identifying mutations in predisposition genes, several studies have been carried out on the association of gene polymorphisms involved in various cellular processes, with the risk and clinicopathological features of BC. Accordingly, we have investigated if some SNPs of *MTHFR*, *XRCC1* and *OGG1* genes affect the pathogenesis in a cohort of Northern Sardinian BC patients and healthy controls.

It is known that folate plays a crucial role in regulating DNA repair and DNA methylation. The *MTHFR* enzyme belongs to a complicated metabolic pathway which also involves other enzymes such as methionine synthase (*MTR*), methionine synthase reductase (*MTRR*). It is biologically plausible that genetic polymorphism of these genes altering the pool of methyl group level can have effects on DNA synthesis, repair and methylation, consequent gene instability and cancer development. *MTHFR* wild-type C genotype can lead to an increased risk due to aberrant DNA methylation leading to activation of proto-oncogenes by hypomethylation of their promoter region and transcriptional silencing of tumor suppressor genes in case of hypermethylation of CpG islands. While the allelic T variant leads to high plasma levels of homocysteine that influences DNA methylation and can lead to changes in the availability of nucleotides for DNA synthesis and repair [44, 45] due to folate-dependent reactions. Balance in these extremely complex processes is therefore strongly influenced not only by gene polymorphisms, but also by the amount of folates introduced through diet, and other factors, including alcohol consumption, and by currently well-known ethnic differences and variable genetic background. For this reason, literature findings show controversial data [46] especially for rs1801133: on the one hand the TT genotype is associated to an increased cancer risk [47–53] on the other hand a relatively large number of studies support a null or protective effect [54–58]. In agreement with the latter, our results have shown that the CT allele of *MTHFR*-rs1801133 is associated with lower risk of BC in the codominant model, while no significant difference was found for the rs1801131 polymorphism in any model examined.

Our study compared to the previous one [23], with the study groups carefully selected regarding ethnicity, greater sample size and with a control group without the auto-selection bias, showed no evidence of association between the *MTHFR*-rs1801133 and rs1801131 and the clinicopathological variables.

Regarding lifestyle variables, some authors report an association between *MTHFR* polymorphisms and some lifestyle approaches such as cigarette smoking status, alcohol intake or high or low folate introduction through diet [59–62]. It is known that cigarette smoking is a major preventable risk factor for lung cancer but also for many other cancers including BC, being able to interfere and modify genetic and epigenetic pathways and the expression of many proinflammatory interleukins [63]. The association between alcohol intake and the risk of developing BC in pre- and postmenopausal women has been demonstrated. Alcohol can act indirectly through the mutagenic and promoting action of its first metabolite acetaldehyde, also by increasing the level of estrogen, causing alterations of the immune system and finally causing nutritional deficiencies including folate and other vitamins [64]. We did not find any significant evidence of interaction between the polymorphisms analyzed and these lifestyles approaches.

It is clear how complex is the study of the relationships existing between genetic factors (we cannot exclude the still unknown interaction with other genes) and lifestyle, especially as regards the folate and alcohol introduction, and higher BC susceptibility. This leads to the conflicting results existing in the literature that can be minimized, within the same population, if the studies are conducted with standardized and rigorous criteria.

It is well-known that gene alterations in components of DNA repair systems, deriving from cumulative exogenous and endogenous mutagens, play a key-role in abnormal growth and malignant transformation. Some common polymorphisms in the gene repair systems may influence the ability of repairing damaged DNA and could be involved in carcinogenesis and tumour progression. The XRCC1 protein plays a central role in BER system, which is responsible for the repair of single-strand DNA break resulting from exposure to X-rays, ROS and alkylating agents [65]. XRCC1 protein acts as a scaffolding protein interacting with 8-oxoguanine DNA glycosylase, DNA ligase III, DNA polymerase β, and poly (ADP-ribose) polymerase at the site of damaged DNA.

In our study no association with the risk of BC was demonstrated for *XRCC1*-rs1799782 (Arg to Trp) and rs25487 (Arg to Gln). The distribution of Arg194Trp and Arg399Gln polymorphisms are significantly influenced by ethnicity: the frequency of the Trp allele is higher in Asian populations than in African and Caucasian populations [66]. Furthermore, it has been observed that patients with the Arg/Arg genotype exhibited significantly higher levels of chromosomal breaks than those with the Trp allele [67]. While the Gln allele of rs25487 leads to a reduction in BER DNA repair activity [24]. Also in this case, the results about the frequency of genotypes of *XRCC1-*rs1799782 (*Arg* to *Trp*) and rs25487 (Arg to Gln) and the BC susceptibility are contrasting [21, 68–77]. In our study, the *XRCC1*-rs25487 polymorphism is nominally associated with family history of BC in two genetic models. In particular, the G/A genotype is significantly associated with sporadic cases in both codominant and overdominant models. Most BC are sporadic forms, which develop in the general population in the absence of familiarity and are mainly related to the action of sex hormones and environmental factors such as smoking, alcohol and nutrition. The transformation into a malignant phenotype depends on the accumulation of random genetic mutations in somatic cells. Such mutations often appear and are/or stabilized during cellular division. The hormones can modulate the growth control mechanisms, cell division frequency, differentiation and, in this way, have the possibility of favoring the onset and accumulation of random genetic errors. In this context, the association we observed, causing as reported a reduction in BER activity, could determine a greater susceptibility to transformation from chemical carcinogens in the mammary gland.

The *OGG1* gene, another component of the BER pathway, is a key enzyme which removes 8-oxoguanine considered as a marker of oxidative stress and highly mutagenic. Our study shows an association between the *OGG1*-rs1052133: the GG (Cys) genotype frequency revealed, indeed a nominal association with lower risk of BC in the recessive model, thus exercising a protective role in BC. In the literature, many functional studies suggest that the 326Cys allele is associated with the reduced DNA repair activity and may contribute to mammary carcinogenesis [78, 79] and other types of cancer [80–82]. However, some studies report conflicting results [74, 83–86]. Weiguang et al. [78] in a meta-analysis study, suggested that the *OGG1* Ser326Cys polymorphism is significantly associated with BC in both premenopausal and postmenopausal European women and the 326*Cys* allele play a protective role in BC carcinogenesis. Synowiec et al. [84] studied the association of polymorphisms of some DNA repair genes with BC by the modulation of the cellular response to oxidative stress. They also observed a protective effect of some genotypes, including the *OGG1* Cys allele, against BC occurrence.

This missense variant has been shown to influence the expression level of *TTLL3* (Tubulin Tyrosine Ligase Like 3) and *OGG1*; more in details, [https://gtexportal.org/home/snp/rs1052133], CC genotype is associated with lower mRNA expression of *TTLL3* in transformed fibroblasts (*p* = 7.4e-8) and tibial artery (*p* = 0.0000034), and with lower expression of *OGG1* in transformed fibroblasts (*p* = 0.000035) [87]. Several previous studies have assessed the role of the *OGG1* polymorphism rs1052133 in human tumors. A statistically significant association was observed between the genotype distribution of rs1052133 and chronic myelogenous leukaemia patients in an African population [88]. The same polymorphism has been nominally associated also with the risk of squamous cell carcinomas of the head and neck (SCCHN) among north Indians [89]; in the same study, the mutant (G) allele has been proposed to be a protective factor for SCCHN among north Indian subpopulations. Furthermore, *OGG1*-rs1052133 polymorphism has been implicated in the pathogenesis of nasopharyngeal carcinoma (NPC), especially among women: genotype GG at rs1052133 was associated with significantly lower NPC risk than genotypes GC + CC OR: 0.770, 95% CI: 0.595–0.996, *p* = 0.012) [90].

Regarding the clinical features, only the *OGG1***-**rs1052133 polymorphism is associated at the nominal level with PgR-status: G allele is significantly more frequent among PgR- cases. Furthermore, it is significantly associated with Her2 status, being more frequent among Her2+ cases. This suggests an association between the G allele and the molecular subtype PgR- and Her2 overexpressing BC in our cohort. Amplification or overexpression of Her-2 occurs in approximately 15–30% of BC. Ali K. et al. [91] in a meta-analysis study showed higher OGG1 mutation frequencies in association with IDC, ER-, PgR-, and HER-2/*neu* status and other clinicopathological features in Pakistani populations and worldwide.

## Conclusion

Our results suggest a possible association between CT genotype in the *MTHFR*-rs1801133 and the GG genotype in the *OGG1*-rs1052133 and a decreasing BC risk in the Northern Sardinia population. Furthermore, some interesting associations were found between some allelic variants and clinical features. However, we believe that latter data should be taken with caution because of the reduction in the sample size that subdivision into sub-categories involves.

Due to its relatively homogeneous genetic composition, the Mediterranean island of Sardinia represents an interesting population for inferring the genetic risk factors in complex diseases such as BC. Here we reported a pilot case-control study carried out on a cohort of Northern Sardinia BC patients and healthy controls, aimed to evaluate the disease risk of genetic variants in three genes involved in DNA methylation and repair mechanisms. Mutational analyses and gene expression profiles allowed us to establish that BC is a complex and heterogeneous disease with marked characteristics of biological variability and response to treatments determined by an equally complex network of aetiological factors. Therefore, it is clear that several studies with larger sample size are warranted both on our and on other ethnic groups to validate the risk factors and to clarify the complex relationships between different genes and their interaction with environmental factors in BC risk and disease progression. Overall, even if our findings should be treated with caution because of the relatively small sample size and heterogeneity, they might be helpful in early detection of BC through identification of population at risk in light of the growing need of decision tools for personalized medicine.

## Supplementary information

**Additional file 1: Table S5** A. Association of MTHFR, XRCC1, OGG1 polymorphisms and ER, PgR, Her-2, Ki67 and Lymph Node status in BC patients. B. Association of MTHFR, XRCC1, OGG1 polymorphisms and Age at diagnosis, BMI, Menopause, BC family history status in BC patients.

## Data Availability

The dataset used and/or analyzed during the current study are available from the corresponding author on reasonable request.

## Abbreviations

BC: Breast cancer
MTHFR: Methylenetetrahydrofolate reductase
XRCC1: X-ray repair cross-complementing group 1
OGG1: 8-Oxoguanine glycosylase
SNP: Single nucleotide polymorphism
BER: Base excision repair
ROS: Reactive oxigen species
BMI: Body mass index
PCR-RFLP: Polymerase chain reaction restriction fragment length polymorphism
HWE: Hardy-Weinberg equilibrium
OD: Odd ratio
CI: Confidence interval
LD: Linkage disequilibrium
IDC: Invasive ductal carcinoma
ER: Estrogen receptor
PgR: Progesterone receptor
