# Supplementary material for: MTHFR, XRCC1 and OGG1 genetic polymorphisms in breast cancer: a case-control study in a population from North Sardinia
Source: BMC Cancer. 2020 Mar 19;20:234. doi: 10.1186/s12885-020-06749-w (PMC7083022; doi:10.1186/s12885-020-06749-w)
Supplement: Supplementary file 1 — Additional file 1: Table S5 A. Association of MTHFR, XRCC1, OGG1 polymorphisms and ER, PgR, Her-2, Ki67 and Lymph Node status in BC patients. B. Association of MTHFR, XRCC1, OGG1 polymorphisms and Age at diagnosis, BMI, Menopause, BC family history status in BC patients. [file 12885_2020_6749_MOESM1_ESM.zip › Suppl_Tab5B Clinical char-lifestR3.docx]

| **Variable** | **Mod** | ***MTHFR* (rs1801133)** | **OR (95% CI)** | **p** | ***MTHFR* (rs1801131)** | **OR (95% CI)** | **p** | ***XRCC1***  **(rs1799782)** | **OR (95% CI)** | **p** | ***XRCC1***  **(rs25487)** | **OR (95% CI)** | **p** | ***OGG1***  **(rs1052133)** | **OR (95% CI)** | **p** |
| --- | --- | --- | --- | --- | --- | --- | --- | --- | --- | --- | --- | --- | --- | --- | --- | --- |
| **Age at diagnosis  > 45/≤45**  **(n.88/47)** | **Co** | C/C 36/17 | 1.00 | - | A/A 42/24 | 1.00 | - | C/C 85/43 | 1.00 | - | G/G 40/24 | 1.00 | - | C/C 56/31 | 1.00 | - |
|  |  | C/T 37/23 | 0.76 (0.35-1.65) | 0.31 | A/C 36/18 | 1.14 (0.54-2.43) | 0.73 | C/T 3/4 | 0.38 (0.08-1.77) | 0.20 | G/A 36/19 | 1.14 (0.54-2.41) | 0.74 | C/G 30/16 | 1.04 (0.49-2.19) | 0.92 |
|  |  | T/T 15/7 | 1.01 (0.35-2.94) | 0.98 | C/C 9/5 | 1.03 (0.31-3.42) | 0.96 |  |  |  | A/A 12/4 | 1.8 (0.52-6.22) | 0.35 | G/G 2/0 | 2.79 (0.13-59.90)* | 0.30 |
|  | **Do** | C/C 36/17 | 1.00 | - | A/A 42/24 | 1.00 | - |  |  |  | G/G 40/24 | 1.00 | - | C/C 56/31 | 1.00 | - |
|  |  | C/T-T/T 52/30 | 0.82 (0.39-1.70) | 0.59 | A/C-C/C 45/23 | 1.11 (0.55-2.27) | 0.76 |  |  |  | G/A-A/A 48/23 | 1.25 (0.62-2.55) | 0.54 | C/G-G/G 32/16 | 1.11 (0.53-2.33) | 0.79 |
|  | **Rec** | C/C-C/T 73/40 | 1.00 | - | A/A-A/C 78/42 | 1.00 | - |  |  |  | G/G-GA 76/43 | 1.00 | - | C/C-C/G 86/47 | 1.00 | - |
|  |  | T/T 15/7 | 1.17 (0.44-3.12) | 0.75 | C/C 9/5 | 0.97 (0.31-3.08) | 0.96 |  |  |  | A/A 12/4 | 1.70 (0.52-5.59) | 0.38 | G/G 2/0 | 2.75 (0.13-58.38)* | 0.30 |
|  | **Ov** | C/C-T/T 51/24 | 1.00 | - | A/A-C/C 51/29 | 1.00 | - |  |  |  | G/G-AA /52/28 | 1.00 | - | C/C-G/G 58/31 | 1.00 | - |
|  |  | C/T 37/23 | 0.76 (0.37-1.54) | 0.44 | A/C 36/18 | 1.14 (0.55-2.35) | 0.73 |  |  |  | G/A 36/19 | 1.02 (0.50-2.10) | 0.96 | C/G 30/16 | 1.00 (0.47-2.12) | 0.99 |
|  | **All** | C 109/57 | 1.00 | - | A 120/66 | 1.00 | - | C 173/9 | 1.00 | - | G 116/67 | 1.00 | - | C 204/78 | 1.00 | - |
|  |  | T 67/37 | 0.95 (0.57-1.58) | 0.84 | C 54/28 | 1.06 (0.61-1.83) | 0.83 | T 3/4 | 0.39 (0.09-1.78) | 0.21 | A 60/27 | 1.28 (0.74-2.21) | 0.37 | G 34/16 | 0.81 (0.42-1.55) | 0.53 |
| **BMI**  **≤25/>25**  **(n.73/62)** | **Co** | C/C 27/26 | 1.00 | - | A/A 33/33 | 1.00 | - | C/C 69/59 | 1.00 | - | G/G 39/25 | 1.00 | - | C/C 46/41 | 1.00 | - |
|  |  | C/T 34/26 | 1.26 (0.60-2.65) | 0.54 | A/C 35/20 | 1.75 (0.84-3.64) | 0.13 | C/T 4/3 | 1.14 (0.25-5.30) | 0.87 | G/A 26/2 | 0.57 (0.28-1.19) | 0.14 | C/G 26/20 | 1.16 (0.56-2.38) | 0.69 |
|  |  | T/T 12/10 | 1.16 (0.43-3.13) | 0.78 | C/C 5/9 | 0.56 (0.17-1.84) | 0.33 |  |  |  | A/A 8/8 | 0.64 (0.21-1.93) | 0.43 | G/G 1/1 | 0.89 (0.05-14.71) | 0.94 |
|  | **Do** | C/C 27/26 | 1.00 | - | A/A 33/33 | 1.00 | - |  |  | - | G/G 39/25 | 1.00 | - | C/C 46/41 | 1.00 | - |
|  |  | C/T-T/T 46/36 | 1.23 (0.62-2.46) | 0.56 | A/C-C/C 40/29 | 1.38 (0.70-2.72) | 0.36 |  |  |  | G/A-A/A 34/37 | 0.59 (0.30-1.17) | 0.13 | C/G-G/G 27/21 | 1.15 (0.56-2.33) | 0.71 |
|  | **Rec** | C/C-C/T 61/52 | 1.00 | - | A/A-A/C 68/53 | 1.00 | - |  |  | - | G/G-G/A 65/54 | 1.00 | - | C/C-C/G 72/61 | 1.00 | - |
|  |  | T/T 12/10 | 1.02 (0.41-2.56) | 0.96 | C/C 5/9 | 0.43 (0.12-1.37) | 0.147 |  |  |  | A/A 8/8 | 0.83 (0.29-2.36) | 0.73 | G/G 1/1 | 0.85 (0.05-13.83) | 0.92 |
|  | **Ov** | C/C-T/T 39/36 | 1.00 | - | A/A-C/C 38/42 | 1.00 | - |  |  | - | G/G-A/A 47/33 | 1.00 | - | C/C-G/G 47/42 | 1.00 | - |
|  |  | C/T 34/26 | 1.21 (0.61-2.39) | 0.59 | A/C 35/20 | 1.93 (0.96-3.91) | 0.07 |  |  |  | G/A 26/29 | 0.63 (0.32-1.26) | 0.19 | C/G 26/20 | 1.16 (0.57-2.38) | 0.68 |
|  | **All** | C 88/78 | 1.00 | - | A 101/86 | 1.00 | - | C 142/121 | 1.00 | - | G 104/79 | 1.00 | - | C 118/102 | 1.00 | - |
|  |  | T 58/46 | 1.12 (0.68-1.8) | 0.66 | C 45/38 | 1.01 (0.60-1.70) | 0.98 | T 4/3 | 1.14 (0.25-5.18) | 0.87 | A 42/45 | 0.71 (0.42-1.18) | 0.19 | G 28/22 | 1.10 (0.59-2.04) | 0.76 |
|  |  |  |  |  |  |  |  |  |  |  |  |  |  |  |  |  |
|  |  |  |  |  |  |  |  |  |  |  |  |  |  |  |  |  |
|  |  |  |  |  |  |  |  |  |  |  |  |  |  |  |  |  |
|  |  |  |  |  |  |  |  |  |  |  |  |  |  |  |  |  |
|  |  |  |  |  |  |  |  |  |  |  |  |  |  |  |  |  |
|  |  |  |  |  |  |  |  |  |  |  |  |  |  |  |  |  |
|  |  |  |  |  |  |  |  |  |  |  |  |  |  |  |  |  |
|  |  |  |  |  |  |  |  |  |  |  |  |  |  |  |  |  |
| **Pre/Post menopause diagnosis**  **(n.72/63)** | **Co** | C/C 28/25 | 1.00 | - | A/A 32/34 | 1.00 | - | C/C 67/61 | 1.00 | - | G/G 36/28 | 1.00 | - | C/C 51/36 | 1.00 | - |
|  |  | C/T 36/24 | 1.34 (0.63-2.83) | 0.44 | A/C 32/23 | 1.48 (0.72-3.04) | 0.29 | C/T 5/2 | 2.28 (0.43-12.17) | 0.33 | G/A 30/25 | 0.93 (0.45-1.93) | 0.85 | C/G 21/25 | 0.59 (0.29-1.22) | 0.15 |
|  |  | T/T 8/14 | 0.51 (0.18-1.42) | 0.20 | C/C 8/6 | 1.42 (0.44-4.53) | 0.56 |  |  |  | A/A 6/10 | 0.47 (0.15-1.44) | 0.18 | G/G 0/2 | 0.14 (0.01-3.04)* | 0.10 |
|  | **Do** | C/C 28/25 | 1.00 | - | A/A 32/34 | 1.00 | - |  |  |  | G/G 36/28 | 1.00 | - | C/C 51/36 | 1.00 | - |
|  |  | C/T-T/T 44/38 | 1.03 (0.52-2.07) | 0.93 | A/C-C/C 40/29 | 1.47 (0.74-2.89) | 0.27 |  |  |  | G/A-A/A 36/35 | 0.8 (0.41-1.58) | 0.52 | C/G-G/G 21/27 | 0.55 (0.27-1.12) | 0.10 |
|  | **Rec** | C/C-C/T 64/49 | 1.00 | - | A/A-A/C 64/57 | 1.00 | - |  |  |  | G/G-G/A 66/53 | 1.00 | - | C/C-C/G 72/61 | 1.00 | - |
|  |  | T/T 8/14 | 0.44 (0.17-1.13) | 0.08 | C/C 8/6 | 1.19 (0.39-3.63) | 0.76 |  |  |  | A/A 6/10 | 0.48 (0.16-1.41) | 0.18 | G/G 0/2 | 0.17 (0.01-3.60)* | 0.13 |
|  | **Ov** | C/C-T/T 36/39 | 1.00 | - | A/A-C/C 40/40 | 1.00 | - |  |  |  | G/G-A/A 42/38 | 1.00 | - | C/C-G/G 51/38 | 1.00 | - |
|  |  | C/T 36/24 | 1.63 (0.82-3.23) | 0.17 | A/C 32/23 | 1.39 (0.70-2.78) | 0.35 |  |  |  | G/A 30/25 | 1.09 (0.55-2.16) | 0.82 | C/G 21/25 | 0.63 (0.31-1.28) | 0.20 |
|  | **All** | C 92/74 | 1.00 | - | A 96/91 | 1.00 | - | C 139/124 | 1.00 | - | G 102/81 | 1.00 | - | C 123/97 | 1.00 | - |
|  |  | T 52/52 | 0.80 (0.49-1.31) | 0.39 | C 48/35 | 1.30 (0.77-2.19) | 0.32 | T 5/2 | 2.23 (0.43-11.70) | 0.33 | A 42/45 | 0.74 (0.44-1.24) | 0.25 | G 21/29 | 0.57 (0.31-1.06) | 0.08 |
| **BC family history Spor/Famil**  **(n.76/59)** | **Co** | C/C 25/28 | 1.00 | - | A/A 40/26 | 1.00 | - | C/C 74/54 | 1.00 | - | G/G 31/33 | 1.00 | - | C/C 50/37 | 1.00 | - |
|  |  | C/T 36/24 | 1.68 (0.80-3.55) | 0.17 | A/C 31/24 | 0.84 (0.41-1.74) | 0.64 | C/T 2/5 | 0.29 (0.05-1.56) | 0.13 | G/A 37/18 | 2.19 (1.04-4.62) | ***0.04*** | C/G 25/21 | 0.88 (0.43-1.81) | 0.73 |
|  |  | T/T 15/7 | 2.4 (0.84-6.84) | 0.10 | C/C 5/9 | 0.36 (0.11-1.20) | 0.09 |  |  |  | A/A 8/8 | 1.06 (0.36-3.18) | 0.91 | G/G 1/1 | 0.74 (0.04-12.22) | 0.83 |
|  | **Do** | C/C 25/28 | 1.00 | - | A/A 40/26 | 1.00 | - |  |  |  | G/G 31/33 | 1.00 | - | C/C 50/37 | 1.00 | - |
|  |  | C/T-T/T 51/31 | 1.84 (0.91-3.71) | 0.09 | A/C-C/C 36/33 | 0.71 (0.36-1.40) | 0.33 |  |  |  | G/A-A/A 45/26 | 1.84 (0.93-3.67) | 0.08 | C/G-G/G 26/22 | 0.87 (0.43-1.78) | 0.71 |
|  | **Rec** | C/C-C/T 61/52 | 1.00 | - | A/A-A/C 71/50 | 1.00 | - |  |  |  | G/G-G/A 68/51 | 1.00 | - | C/C-C/G 75/58 | 1.00 | - |
|  |  | T/T 15/7 | 1.83 (0.69-4.82) | 0.22 | C/C 5/9 | 0.39 (0.12-1.24) | 0.10 |  |  |  | A/A 8/8 | 0.75 (0.26-2.13) | 0.59 | G/G 1/1 | 0.77 (0.05-12.63) | 0.86 |
|  | **Ov** | C/C-T/T 40/35 | 1.00 | - | A/A-C/C 45/35 | 1.00 | - |  |  |  | G/G-A/A 39/41 | 1.00 | - | C/C-G/G 51/38 | 1.00 | - |
|  |  | C/T 36/24 | 1.3 (0.66-2.61) | 0.44 | A/C 31/24 | 1.00 (0.50-2.01) | 0.99 |  |  |  | G/A 37/18 | 2.16 (1.06-4.41) | ***0.03*** | C/G 25/21 | 0.89 (0.43-1.81) | 0.74 |
|  | **All** | C 86/80 | 1.00 | - | A 111/76 | 1.00 | - | C 150/113 | 1.00 | - | G 99/84 | 1.00 | - | C 125/95 | 1.00 | - |
|  |  | T 66/38 | 1.62 (0.98-2.67) | 0.06 | C 41/42 | 0.67 (0.40-1.12) | 0.13 | T 2/5 | 0.30 (0.06-1.58) | 0.13 | A 53/34 | 1.32 (0.79-2.22) | 0.29 | G 27/23 | 0.89 (0.48-1.65) | 0.72 |

**Table 5B.** Association of *MTHFR*, *XRCC1*, *OGG1* polymorphisms and Age at diagnosis, BMI, Menopause, BC family history status in BC patients.

*Gart adjusted logit interval
