# Supplementary material for: MTHFR, XRCC1 and OGG1 genetic polymorphisms in breast cancer: a case-control study in a population from North Sardinia
Source: BMC Cancer. 2020 Mar 19;20:234. doi: 10.1186/s12885-020-06749-w (PMC7083022; doi:10.1186/s12885-020-06749-w)
Supplement: Supplementary file 1 — Additional file 1: Table S5 A. Association of MTHFR, XRCC1, OGG1 polymorphisms and ER, PgR, Her-2, Ki67 and Lymph Node status in BC patients. B. Association of MTHFR, XRCC1, OGG1 polymorphisms and Age at diagnosis, BMI, Menopause, BC family history status in BC patients. [file 12885_2020_6749_MOESM1_ESM.zip › Suppl_Tab5A_ClinicopatholR3.docx]

| **Variable** | **Model** | ***MTHFR***  **(rs1801133)** | **OR (95% CI)** | **p** | ***MTHFR***  **(rs1801131)** | **OR (95% CI)** | **p** | ***XRCC1***  **(rs1799782)** | **OR (95% CI)** | **p** | ***XRCC1***  **(rs25487)** | **OR (95% CI)** | **p** | ***OGG1***  **rs1052133** | **OR (95% CI)** | **p** |
| --- | --- | --- | --- | --- | --- | --- | --- | --- | --- | --- | --- | --- | --- | --- | --- | --- |
| **ER+/ER-**  **(n. 97/38)** | **Co** | C/C 40/13 | 1.00 | - | A/A 50/16 | 1.00 | - | C/C 90/38 | 1.00 | - | G/G 50/14 | 1.00 | - | C/C 63/24 | 1.00 | - |
|  |  | C/T 40/20 | 0.65 (0.29-1.48) | 0.31 | A/C 37/18 | 0.66 (0.30-1.46) | 0.30 | C/T 7/0 | 6.32 (0.36-114.53) | 0.09 | G/A 35/20 | 0.49 (0.22-1.10) | 0.08 | C/G 33/13 | 0.97 (0.44-2.14) | 0.93 |
|  |  | T/T 17/5 | 1.11 (0.34-3.59) | 0.87 | C/C 10/4 | 0.8 (0.22-2.90) | 0.74 |  |  |  | A/A 12/4 | 0.84 (0.23-3.01) | 0.79 | G/G 1/1 | 0.38 (0.02-6.33) | 0.49 |
|  | **Do** | C/C 40/13 | 1.00 | - | A/A 50/16 | 1.00 | - |  |  |  | G/G 50/14 | 1.00 | - | C/C 63/24 | 1.00 | - |
|  |  | C/T-T/T 57/25 | 0.74 (0.34-1.62) | 0.45 | A/C-C/C 47/22 | 0.68 (0.32-1.46) | 0.33 |  |  |  | G/A-A/A 47/24 | 0.55 (0.25-1.18) | 0.13 | C/G-G/G 34/14 | 0.93 (0.42-2.02) | 0.85 |
|  | **Rec** | C/C-C/T 80/33 | 1.00 | - | A/A-A/C 87/34 | 1.00 | - |  |  |  | G/G-G/A 85/34 | 1.00 | - | C/C-C/G 96/37 | 1.00 | - |
|  |  | T/T 17/5 | 1.40 (0.48-4.12) | 0.54 | C/C 10/4 | 0.98 (0.29-3.33) | 0.97 |  |  |  | A/A 12/4 | 1.2 (0.36-3.98) | 0.77 | G/G 1/1 | 0.39 (0.02-6.32) | 0.49 |
|  | **Ov** | C/C-T/T 57/18 | 1.00 | - | A/A-C/C 60/20 | 1.00 | - |  |  |  | G/G-A/A 62/18 | 1.00 | - | C/C-G/G 64/25 | 1.00 | - |
|  |  | C/T 40/20 | 0.63 (0.30-1.34) | 0.23 | A/C 37/18 | 0.69 (0.32-1.5) | 0.33 |  |  |  | G/A 35/20 | 0.51 (0.24-1.09) | 0.08 | C/G 33/13 | 0.99 (0.45-2.19) | 0.98 |
|  | **All** | C 120/46 | 1.00 | - | A 137/50 | 1.00 | - | C 187/76 | 1.00 | - | G 135/48 | 1.00 | - | C 159/61 | 1.00 | - |
|  |  | T 74/30 | 0.95 (0.55-1.63) | 0.84 | C 57/26 | 0.80 (0.45-1.41) | 0.44 | T 7/0 | 6.12 (0.35-108.49) | 0.09 | A 59/28 | 0.75 (0.43-1.31) | 0.31 | G 35/15 | 0.90 (0.46-1.75) | 0.75 |
| **PgR+/PgR-**  **(n. 90/45)** | **Co** | C/C 37/16 | 1.00 | - | A/A 47/19 | 1.00 | - | C/C 85/43 | 1.00 | - | G/G 42/22 | 1.00 | - | C/C 58/29 | 1.00 | - |
|  |  | C/T 35/25 | 0.61 (0.28-1.32) | 0.21 | A/C 35/20 | 0.71 (0.33-1.52) | 0.38 | C/T 5/2 | 1.26 (0.24-6.79) | 0.78 | G/A 36/19 | 0.99 (0.47-2.12) | 0.98 | C/G 32/14 | 1.14 (0.53-2.47) | 0.74 |
|  |  | T/T 18/4 | 1.95 (0.57-6.67) | 0.29 | C/C 8/6 | 0.54 (0.16-1.77) | 0.31 |  |  |  | A/A 12/4 | 1.57 (0.45-5.45) | 0.48 | G/G 0/2 | 0.10 (0.05-2.16)* | 0.05 |
|  | **Do** | C/C 37/16 | 1.00 | - | A/A 47/19 | 1.00 | - |  |  |  | G/G 42/22 | 1.00 | - | C/C 58/29 | 1.00 | - |
|  |  | C/T-T/T 53/29 | 0.79 (0.38-1.66) | 0.53 | A/C-C/C 43/26 | 0.67 (0.32-1.4) | 0.28 |  |  |  | G/A-A/A 48/23 | 1.09 (0.53-2.24) | 0.81 | C/G-G/G 32/16 | 1 (0.47-2.11) | 1.00 |
|  | **Rec** | C/C-C/T 72/41 | 1.00 | - | A/A-A/C 82/39 | 1.00 | - |  |  |  | G/G-G/A 78/41 | 1.00 | - | C/C-C/G 90/43 | 1.00 | - |
|  |  | T/T 18/4 | 2.56 (0.81-8.09) | 0.10 | C/C 8/6 | 0.63 (0.21-1.95) | 0.43 |  |  |  | A/A 12/4 | 1.58 (0.48-5.20) | 0.45 | G/G 0/2 | 0.09 (0.04-2.04)* | ***0.04*** |
|  | **Ov** | C/C-T/T 55/20 | 1.00 | - | A/A-C/C 55/25 | 1.00 | - |  |  |  | G/G-A/A 54/26 | 1.00 | - | C/C-G/G 58/31 | 1.00 | - |
|  |  | C/T 35/25 | 0.51 (0.25-1.05) | 0.07 | A/C 35/20 | 0.80 (0.39-1.64) | 0.54 |  |  |  | G/A 6/19 | 0.91 (0.44-1.89) | 0.48 | C/G 32/14 | 1.22 (0.57-2.62) | 0.61 |
|  | **All** | C 109/57 | 1.00 | - | A 129/58 | 1.00 | - | C 175/88 | 1.00 | - | G 120/63 | 1.00 | - | C 148/72 | 1.00 | - |
|  |  | T 71/33 | 1.13 (0.67-1.90) | 0.66 | C 51/32 | 0.72 (0.42-1.23) | 0.23 | T 5/2 | 1.26 (0.24-6.61) | 0.79 | A 60/27 | 1.17 (0.68-2.02) | 0.58 | G 32/18 | 0.86 (0.45-1.64) | 0.66 |
| **Her2+/Her2 (n. 42/88)** | **Co** | C/C 17/36 | 1.00 | - | A/A 21/42 | 1.00 | - | C/C 40/84 | 1.00 | - | G/G 18/45 | 1.00 | - | C/C 30/52 | 1.00 | - |
|  |  | C/T 15/41 | 0.77 (0.34-1.77) | 0.55 | A/C 18/35 | 1.03 (0.47-2.23) | 0.94 | C/T 2/4 | 1.05 (0.18-5.97) | 0.96 | G/A 17/34 | 1.25 (0.56-2.78) | 0.63 | C/G 10/36 | 0.48 (0.21-1.11) | 0.08 |
|  |  | T/T 10/11 | 1.93 (0.69-5.41) | 0.21 | C/C 3/11 | 0.55 (0.14-2.17) | 0.39 |  |  |  | A/A 7/9 | 1.94 (0.63-6.01) | 0.25 | G/G 2/0 | 8.61 (0.40-185.22)* | 0.07 |
|  | **Do** | C/C 17/36 | 1.00 | - | A/A 21/42 | 1.00 | - |  |  |  | G/G 18/45 | 1.00 | - | C/C 30/52 | 1.00 | - |
|  |  | C/T-T/T 25/52 | 1.02 (0.48-2.15) | 0.96 | A/C-C/C 21/46 | 0.91 (0.44-1.90) | 0.81 |  |  |  | G/A-A/A 24/43 | 1.40 (0.67-2.93) | 0.38 | C/G-G/G 12/36 | 0.58 (0.26-1.28) | 0.17 |
|  | **Rec** | C/C-C/T 32/77 | 1.00 | - | A/A-A/C 39/77 | 1.00 | - |  |  |  | G/G-G/A 35/79 | 1.00 | - | C/C-C/G 40/88 | 1.00 | - |
|  |  | T/T 10/11 | 2.19 (0.85-5.66) | 0.10 | C/C 3/11 | 0.54 (0.14-2.04) | 0.36 |  |  |  | A/A 7/9 | 1.76 (0.61-5.10) | 0.30 | G/G 2/0 | 10.92 (0.51-232.81)* | ***0.04*** |
|  | **Ov** | C/C-T/T 27/47 | 1.00 | - | A/A-C/C 24/53 | 1.00 | - |  |  |  | G/G-A/A 25/54 | 1.00 | - | C/C-G/G 32/52 | 1.00 | - |
|  |  | C/T 15/41 | 0.64 (0.30-1.36) | 0.24 | A/C 18/35 | 1.14 (0.54-2.39) | 0.74 |  |  |  | G/A 17/34 | 1.08 (0.51-2.29) | 0.84 | C/G 10/36 | 0.45 (0.20-1.03) | 0.06 |
|  | **All** | C 49/113 | 1.00 | - | A 60/119 | 1.00 | - | C 82/172 | 1.00 | - | G 53/124 | 1.00 | - | C 70/140 | 1.00 | - |
|  |  | T 35/63 | 1.28 (0.75-2.18) | 0.36 | C 24/57 | 0.84 (0.47-1.48) | 0.54 | T 2/4 | 1.05 (0.18-5.97) | 0.96 | A 31/52 | 1.39 (0.81-2.41) | 0.23 | G 14/36 | 0.78 (0.39-1.54) | 0.47 |
|  |  |  |  |  |  |  |  |  |  |  |  |  |  |  |  |  |
|  |  |  |  |  |  |  |  |  |  |  |  |  |  |  |  |  |
|  |  |  |  |  |  |  |  |  |  |  |  |  |  |  |  |  |
|  |  |  |  |  |  |  |  |  |  |  |  |  |  |  |  |  |
|  |  |  |  |  |  |  |  |  |  |  |  |  |  |  |  |  |
|  |  |  |  |  |  |  |  |  |  |  |  |  |  |  |  |  |
|  |  |  |  |  |  |  |  |  |  |  |  |  |  |  |  |  |
| **Ki67+/Ki67- (n. 45/89)** | **Co** | C/C 16/37 | 1.00 | - | A/A 26/39 | 1.00 | - | C/C 42/85 | 1.00 | - | G/G 21/43 | 1.00 | - | C/C 30/57 | 1.00 | - |
|  |  | C/T 21/38 | 1.28 (0.58-2.82) | 0.55 | A/C 14/41 | 0.51 (0.23-1.12) | 0.09 | C/T 3/4 | 1.52 (0.32-7.09) | 0.59 | G/A 16/39 | 0.84 (0.38-1.84) | 0.66 | C/G 14/31 | 0.86 (0.40-1.85) | 0.70 |
|  |  | T/T 8/14 | 1.32 (0.46-3.77) | 0.60 | C/C 5/9 | 0.83 (0.25-2.77) | 0.77 |  |  |  | A/A 8/7 | 2.34 (0.75-7.32) |  | G/G 1/1 | 1.90 (0.11-31.46) | 0.65 |
|  | **Do** | C/C 16/37 | 1.00 | - | A/A 26/39 | 1.00 | - |  |  |  | G/G 21/43 | 1.00 | - | C/C 30/57 | 1.00 | - |
|  |  | C/T-T/T 29/52 | 1.29 (0.61-2.71) | 0.50 | A/C-C/C 19/50 | 0.57 (0.28-1.18) | 0.13 |  |  |  | G/A-A/A 24/46 | 1.07 (0.52-2.19) | 0.10 | C/G-G/G 15/32 | 0.89 (0.42-1.90) | 0.77 |
|  | **Rec** | C/C-C/T 37/75 | 1.00 | - | A/A-A/C 40/80 | 1.00 | - |  |  |  | G/G-G/A 37/82 | 1.00 | - | C/C-C/G 44/88 | 1.00 | - |
|  |  | T/T 8/14 | 1.16 (0.45-3.01) | 0.76 | C/C 5/9 | 1.11 (0.35-3.53) | 0.86 |  |  |  | A/A 8/7 | 2.53 (0.85-7.50) | 0.09 | G/G 1/1 | 2.00 (0.12-32.74) | 0.62 |
|  | **Ov** | C/C-T/T 24/51 | 1.00 | - | A/A-C/C 31/48 | 1.00 | - |  |  |  | G/G-A/A 29/50 | 1.00 | - | C/C-G/G 31/58 | 1.00 | - |
|  |  | C/T 21/38 | 1.17 (0.57-2.41) | 0.66 | A/C 14/41 | 1.53 (0.25-1.13) | 0.17 |  |  |  | G/A 16/39 | 0.71 (0.34-1.48) | 0.36 | C/G 14/31 | 0.85 (0.39-1.82) | 0.67 |
|  | **All** | C 53/112 | 1.00 | - | A 66/119 | 1.00 | - | C 87/174 | 1.00 | - | G 58/125 | 1.00 | - | C 74/145 | 1.00 | - |
|  |  | T 37/66 | 1.18 (0.71-1.99) | 0.52 | C 24/59 | 0.73 (0.42-1.29) | 0.28 | T 3/4 | 1.50 (0.33-6.85) | 0.60 | A 32/53 | 1.30 (0.77-2.23) | 0.34 | G 16/33 | 0.95 (0.49-1.84) | 0.88 |
| **Lymph Nodes Neg/Pos**  **(n. 59/66)** | **Co** | C/C 23/27 | 1.00 | - | A/A 30/32 | 1.00 | - | C/C 56/62 | 1.00 | - | G/G 26/32 | 1.00 | - | C/C 35/44 | 1.00 | - |
|  |  | C/T 24/30 | 0.93 (0.43-2.03) | 0.87 | A/C 21/28 | 0.80 (0.38-1.70) | 0.56 | C/T 3/4 | 0.830 (0.18-3.87) | 0.81 | G/A 25/26 | 1.18 (0.56-2.52) | 0.66 | C/G 24/20 | 1.51 (0.72-3.17) | 0.28 |
|  |  | T/T 12/9 | 1.57 (0.56-4.37) | 0.39 | C/C 8/6 | 1.42 (0.44-4.58) | 0.56 |  |  |  | A/A 8/7 | 1.41 (0.45-4.39) | 0.56 | G/G 0/2 | 0.25 (0.01-5.39)* | 0.22 |
|  | **Do** | C/C 23/27 | 1.00 | - | A/A 30/32 | 1.00 | - |  |  |  | G/G 26/32 | 1.00 | - | C/C 35/44 | 1.00 | - |
|  |  | C/T-T/T 36/39 | 1.08 (0.53-2.22) | 0.82 | A/C-C/C 29/34 | 0.91 (0.45-1.84) | 0.79 |  |  |  | G/A-A/A 33/33 | 1.23 (0.61-2.50) | 0.57 | C/G-G/G 24/22 | 1.37 (0.66-2.84) | 0.40 |
|  | **Rec** | C/C-C/T 47/57 | 1.00 | - | A/A-A/C 51/60 | 1.00 | - |  |  |  | G/G-G/A 51/58 | 1.00 | - | C/C-C/G 59/64 | 1.00 | - |
|  |  | T/T 12/9 | 1.62 (0.63-4.17) | 0.32 | C/C 8/6 | 1.57 (0.51-4.82) | 0.43 |  |  |  | A/A 8/7 | 1.30 (0.44-3.83) | 0.64 | G/G 0/2 | 0.22 (0.01-4.61)* | 0.18 |
|  | **Ov** | C/C-T/T 35/36 | 1.00 | - | A/A-C/C 38/38 | 1.00 | - |  |  |  | G/G-A/A 34/39 | 1.00 | - | C/C-G/G 35/46 | 1.00 | - |
|  |  | C/T 24/30 | 0.82 (0.40-1.67) | 0.59 | A/C 21/28 | 0.75 (0.36-1.55) | 0.44 |  |  |  | G/A 25/26 | 1.10 (0.54-2.26) | 0.79 | C/G 24/20 | 1.58 (0.75-3.30) | 0.23 |
|  | **All** | C 70/84 | 1.00 | - | A 81/92 | 1.00 | - | C 115/128 | 1.00 | - | G 77/90 | 1.00 | - | C 94/108 | 1.00 | - |
|  |  | T 48/48 | 1.2 (0.72-1.99) | 0.48 | C 37/40 | 1.05 (0.61-1.80) | 0.86 | T 3/4 | 0.83 (0.18-3.81) | 0.82 | A 41/40 | 1.20 (0.70-2.04) | 0.51 | G 24/24 | 1.15 (0.61-2.16) | 0.67 |

**Table 5A.** Association of *MTHFR*, *XRCC1*, *OGG1* polymorphisms and ER, PgR, Her-2, Ki67 and Lymph Node status in BC patients.

*Gart adjusted logit interval
